# Modulation of the tumor microenvironment by armed vesicular stomatitis virus in a syngeneic pancreatic cancer model

**DOI:** 10.1186/s12985-022-01757-7

**Published:** 2022-02-23

**Authors:** Sijia Tang, Lei Shi, Breona T. Luker, Channen Mickler, Bhavana Suresh, Gregory B. Lesinski, Daping Fan, Yuan Liu, Ming Luo

**Affiliations:** 1 Institute of Biomedical Sciences, Georgia State University, Atlanta, GA 30302 USA; 2 Department of Biology, Georgia State University, Atlanta, GA 30302 USA; 3 Department of Chemistry, Georgia State University, Atlanta, GA 30302 USA; 4 Department of Hematology and Medical Oncology, Winship Cancer Institute of Emory University, Atlanta, GA 30322 USA; 5 Department of Cell Biology and Anatomy, University of South Carolina School of Medicine, Columbia, SC 29209 USA; 6 Center for Diagnostics and Therapeutics, Georgia State University, Atlanta, GA 30302 USA

**Keywords:** Smac, Tumor microenvironment, Neutrophil, MDSC, M2 macrophages

## Abstract

**Background:**

The immunosuppressive microenvironment in pancreatic ductal adenocarcinoma is a major factor that limits the benefits of immunotherapy, especially immune checkpoint blockade. One viable strategy for reverting the immunosuppressive conditions is the use of an oncolytic virus (OV) in combination with other immunotherapy approaches. Infection of PDAC cells with a robust OV can change the tumor microenvironment and increase tumor antigen release by its lytic activities. These changes in the tumor may improve responses to immunotherapy, including immune checkpoint blockade. However, a more potent OV may be required for efficiently infecting pancreatic tumors that may be resistant to OV.

**Methods:**

Vesicular stomatitis virus, a rapid replicating OV, was armed to express the Smac protein during virus infection (VSV-S). Adaptation by limited dilution largely increased the selective infection of pancreatic cancer cells by VSV-S. The engineered OV was propagated to a large quantity and evaluated for their antitumor activities in an animal model.

**Results:**

In a syngeneic KPC model, intratumoral injection of VSV-S inhibited tumor growth, and induced increasing tumor infiltration of neutrophils and elimination of myeloid derived suppressor cells and macrophages in the tumor. More importantly, M2-like macrophages were eliminated preferentially over those with an M1 phenotype. Reduced levels of arginase 1, TGF-β and IL-10 in the tumor also provided evidence for reversion of the immunosuppressive conditions by VSV-S infection. In several cases, tumors were completely cleared by VSV-S treatment, especially when combined with anti-PD-1 therapy. A long-term survival of 44% was achieved.

**Conclusions:**

The improved OV, VSV-S, was shown to drastically alter the immune suppressive tumor microenvironment when intratumorally injected. Our results suggest that the combination of potent OV treatment with immune checkpoint blockade may be a promising strategy to treat pancreatic cancer more effectively.

## Background

Pancreatic Ductal Adenocarcinoma (PDAC) represents ~ 90% of all pancreatic cancers [1]. While surgery can be curative in some PDAC patients when disease is diagnosed at early stage, most PDAC patients present with metastasis or are not operable at the time of diagnosis due to locally advanced disease [2, 3]. Several front-line chemotherapies used as the standard of care for advanced and metastatic PDAC resulting in overall survival benefits measuring only in months [4, 5]. Key mutations in oncogenes and tumor suppressor genes have been identified in PDAC including Kirsten-ras protein (KRAS), cyclin-dependent kinase inhibitor 2A (CDKN2A), tumor protein p53 (TP53) and mothers against decapentaplegic homolog 4 (SMAD4) [6]. However, targeting these pathways is plagued by resistance and limited availability of useful targeted agents. Given these factors, improved treatment strategies for PDAC are urgently needed. Gemcitabine is the most commonly used drug since 1997. More recently, combination therapy of 5-fluoruracil (5-FU), leucovorin (LV), irinotecan and oxaliplatin (FOLFIRINOX) and gemcitabine-nab-paclitaxel significantly increased the median overall survival in comparison with gemcitabine [7]. A large number trials with different combination therapies, with addition of drugs targeting new pathways, have not yet demonstrated further improvement [8]. These efforts are ongoing with addition of more targeted therapies. Due to the complicated genetic plasticity of PDAC, it is very hard to design an effective combination therapy. While improvements along these strategies are likely to continue, several new approaches have emerged lately.

To date, immunotherapy has also shown limited success in PDAC patients [9]. This limited efficacy is a consequence of multiple factors. First, an intense stromal desmoplastic reaction can limit access of cells into tumors. This occurs possibly both by physical barriers due to excess collagen production and via secretion of soluble factors, including CXCL12, that prevent proper T cell chemotaxis [10]. Second, many cells within the tumor microenvironment (TME) serve as a source of immunosuppressive cytokines including interleukin-6 (IL-6) and transforming growth factor-β (TGF-β) that further inhibit antitumor immune responses via redundant mechanisms [11–13]. Third, abundant suppressive immune cells including T regulatory cells (T reg), myeloid derived suppressor cells (MDSC) are also present that further maintain “immune privilege” [14–16]. Finally, PDAC tumors have a lower frequency of somatic mutations, which in theory limits the abundance of neoantigens available for promoting immune response [17]. To circumvent these multiple limitations, combination therapies consisting of chemotherapy or radiation with immunotherapy are currently under investigation in several clinical trials for patients with PDAC [18].

A newly emerging therapy using oncolytic virus (OV) has shown improvements on the efficacy of immunotherapy in pre-clinical models [19, 20]. OV can change the TME to enhance anti-tumor immunity and releases novel neoantigens through its oncolysis. It was shown that tumor-associated macrophages (TAMs), especially the anti-inflammatory macrophages, were downregulated and increased the percentage of tumor-infiltrating lymphocytes. Activated cytotoxic CD8 + T cells and T helper (Th)1 cells were increased by treatment of OV herpes simplex virus-1 in the syngeneic PDAC model, based on single cell RNA sequencing (scRNA-seq) and multicolor fluorescence-activated cell sorting (FACS) analysis [19]. A significant enhancement of tumor-specific IFNγ production was observed by restimulation with growth-arrested tumor cells of splenocytes isolated from murine pancreatic DT6606 subcutaneous tumors treated with a novel oncolytic Vaccinia virus [21]. As more evidence for this, the efficacy of anti-PD-1 immunotherapy in treatment of patients with advanced melanoma was enhanced when combined with talimogene laherparepvec (T-Vec) [22]. Patients who responded to combination therapy had increased CD8 + T cells, and elevated PD-L1 protein expression in tumors. We have devised an armed oncolytic virus, VSV-S, that has an expression cassette for Smac inserted in the genome of vesicular stomatitis virus (VSV). Smac is a mitochondrial protein that mitigates a class of negative regulators of apoptosis, known as the inhibitor of apoptosis proteins (IAPs), when Smac is released in the cytosol. The endogenous Smac was diminished by infection of wt VSV [23]. VSV-S expressed a high level of Smac to replenish the endogenous Smac and induced elevated apoptosis via the caspase-9 pathway and strong tumor necrosis in a human breast cancer model in nude mice, and inhibition of tumor growth in the syngeneic mouse model [23]. We report here the change of the TME induced by VSV-S in the KPC-based mouse model of pancreatic cancer, and demonstrate the combined treatment with VSV-S and anti-PD-1 antibody has significant growth inhibition as compared to either agent alone.

## Materials and methods

### Cell, virus and antibody

*Cells* HeLa, MS1 and MIA PaCa-2 cells were purchased from ATCC. MS1 is a mouse pancreatic islet endothelial cell line. KPC_Luc cells were obtained from Dr. Craig Logsdon (MD Anderson Cancer Center). Cells except for MS1 were grown in DMEM, supplemented with 10% Fetal Bovine Serum (FBS), at 37 °C, 5% CO_2_. MS1 cells were grown in DMEM, supplemented with 5% Fetal Bovine Serum (FBS), at 37 °C, 5% CO_2_.

*Viruses* VSV-S and wt VSV were generated by reverse genetics as described previously [23]. Virus stocks were grown in HeLa cells maintained in DMEM without FBS and stored in liquid nitrogen. VSV-S_KPC_ was grown in KPC_Luc cells in DMEM, supplemented with 2% FBS. Concentrated VSV-S_KPC_ was resuspended in PBS with 5% sucrose, and stored in liquid nitrogen.

*Antibodies* anti-PD-1 (mouse) was purchased from BioXcell (Clone: RMP1-14, catalog #: BE0146). Antibodies used for flow cytometry and immunohistochemistry staining including pacific blue-conjugated rat-anti-mouse CD45 (Clone: 30-F11, catalog #: 103126), FITC-conjugated rat-anti-mouse CD11b (Clone: M1/70, catalog #: 101206), pacific blue-conjugated rat-anti-mouse CD11b(Clone: M1/70, catalog #: 101224), FITC-conjugated rat-anti-mouse Ly6C (Clone: HK1.4, catalog #: 128006), Brilliant Violet 650-conjugated rat-anti-mouse F4/80 (Clone: BM8, catalog #: 123149), PE/Cyanine7-conjugated rat-anti-mouse Ly6G (Clone: 1A8, catalog #: 127618), PE/Cyanine7-conjugated rat-anti-mouse CD8a (Clone: 53-6.7, catalog #: 100722) and PE-conjugated rat-anti-mouse CD4 (Clone: RM4-5, catalog #: 100512) were purchased from BioLegend® Inc. (San Diego, CA).

### Animals

All animal studies followed the protocol approved by GSU IACUC. C57BL/6 mice (male and female, 6 week old) were purchased from Jackson Laboratory. Tumors were implanted by subcutaneous injection of 0.5 × 10^6^ KPC_Luc cells in the flank of each mouse. The overall tumor burden was recorded by measuring the luciferase activity. For these studies, 100 µL of a luciferin solution, 15 mg/mL in PBS, was injected intraperitoneally in each mouse, and mice were imaged in IVIS Spectrum Imager (PerkinElmer) 10 min after injection of luciferin.

### Flow cytometry

Flow cytometry was carried out as described in Bian et al. [24]. Briefly, tumors were isolated from the mice and digested into single cells with the GentleMACS Dissociator (Miltenyi biotec, Germany). To improve recovery of macrophages and other myeloid leukocytes, the trypsin was added, followed by red blood cell lysis. For staining, cells were incubated in Fc blocker (Bio X Cell, NH) for 10 min at room temperature, followed by incubating with the fluorophore-conjugated antibodies cocktail for 30 min at 4 °C. Dead cells were excluded by 7-AAD staining. The tumor-associated leukocytes are gated based on their expression of lineage defining markers (e.g., CD45 for leukocytes, CD45 + CD11b + F4/80 + Ly6C^high^ for monocytes). For each sample, 300,000 events were collected by LSR Fortessa (BD Bioscience) flow cytometer. The results were analyzed by using FlowJo (Becton Dickinson, OR).

### Immunohistochemistry staining

After the mice were sacrificed, the tumors were isolated and fixed in 10% formalin for 48 h in room temperature. The tumors were embedded in paraffin and serial sections (4 µm in thickness). For immunohistochemistry (IHC) assays, slides were deparaffinized, soaked in an antigen retrieval buffer, and steamed for 40 min for antigen retrieving. The endogenous peroxidase activity was quenched with 3% hydrogen peroxide in 10% PBS for 10 min. The nonspecific binding sites were blocked with protein block (Biogenex, CA) at room temperature for 30 min. The slides were incubated with primary antibodies diluted in TBS with 1% BSA at 37 °C for 1 h, and then with the secondary antibody (Dako, Denmark) at room temperature for 30 min. The slides were then stained with diaminobenzidine and counterstained with hematoxylin. Images of stained tissue sections were recorded under AxioVert 40 CFL Image system (Carl Zeiss, Germany). The results were analyzed by using a quantitative image analysis system of the ImageJ software version 1.53e (National Institutes of Health, MD). A mean value was determined from at least ten sections from each tumor.

### MTT assay

MTT assays were carried out using the kit CellTiter 96® from Promega. Briefly, 15 µL of Dye solution was added to 100 µL of PBS in each well of cells (0.5 × 10^6^) that were infected at different MOI for 24 h. After 1 h incubation at 37 °C, 5% CO_2_, 100 µL of Stop solution was added in each well. After overnight gentle shaking, the absorbance was measured at 570 nm.

### Safety study

Hematology, Clinical Chemistry and Coagulation assays were carried by IDEXX BioAnalytics (6006 Comprehensive Chemistry, 6005 Coagulation Mini).

### Statistical methods

Student t-test of two-tails was performed for comparisons of data from flow cytometry, immunohistochemistry staining and tumor growth. Marks are: * stands for *p* < 0.05, ** stands for *p* < 0.01, *** stands for *p* < 0.001, **** stands for *p* < 0.0001.

## Results

### VSV-S adaptation by limited dilution

VSV is a negative strand RNA virus that has a broad cellular tropism. The host receptor for VSV is the LDL receptor and its family members [25]. VSV is commonly propagated in established cell lines, including HeLa and Vero cells. Since the mutation rate of VSV is high, it can be adapted to selected cell types by serial passaging [26]. To increase the infectivity targeting PDAC cells, VSV-S was adapted by limited dilution.

Recombinant VSV-S was previously constructed [23]. A stock of VSV-S was prepared by infecting HeLa cells. The titer of VSV-S in the culture medium of the infected HeLa cells was 1.5 ± 0.15 × 10^6^ PFU/mL (Table 1). A set of VSV-S inocula was prepared by tenfold dilution (10^–1^ to 10^–6^). The same set of inocula was also prepared for a wt VSV that expresses a viral P protein fused with mCherry as a control to monitor cell infection. MIA PaCa-2 cells, a human pancreatic cancer cell line, were infected in individual wells with the two dilution sets of virus inocula. Cytopathogenic effect (CPE) was examined under a microscope 24 h post-infection. The dilution at which MIA PaCA-2 cells were effectively infected by VSV-S was selected based on the level of CPE that was the same as for the fluorescent wt VSV. The culture medium from the well infected with the inoculum at the selected dilution was collected and was used to prepare the next set of tenfold diluted VSV inocula. This process was repeated a total of 5 rounds. The virus sample (designated as VSV-S_PC_) from the 5th round of adaptation was characterized by the plaque assays using HeLa cells and by TCID50 determination using MIA PaCa-2 cells. The results are summarized in Table 1, in comparison with VSV-S propagated in HeLa cells. The titer of VSV- S_PC_ adapted in MIA PaCa-2 cells was increased by 100-fold in comparison with unadapted VSV-S.

**Table 1:** Titers of MIA adapted VSV-S

	VSV-S_PC_	VSV-S
PFU/mL (HeLa)	1.6 ± 0.2 × 10^8^	1.5 ± 0.15 × 10^6^
TCID50 (MIA PaCa-2)	3.9 ± 0.8 × 10^8^	3.6 ± 0.6 × 10^6^

By the same method, VSV-S was also adapted a murine PC cell line, KPC, that was derived from the Kras^G12D^; Trp53^R172H^; Pdx1-Cre (KPC) mouse model. The adaption by limited dilution was carried out by 5 rounds in KPC cells. Comparisons of VSV-SKPC versus VSV-S propagated in HeLa cells are summarized in Table 2. The data suggested that adaptation increased the titer of VSV-S adapted in KPC cells by at least 20-fold.

**Table 2:** Titers of KPC adapted VSV-S

	VSV-S_KPC_	VSV-S
PFU/mL (HeLa)	3.3 ± 0.7 × 10^7^	1.5 ± 0.15 × 10^6^
TCID50 (KPC)	6.3 ± 1.1 × 10^7^	3.4 ± 0.6 × 10^6^

**Fig. 1:**
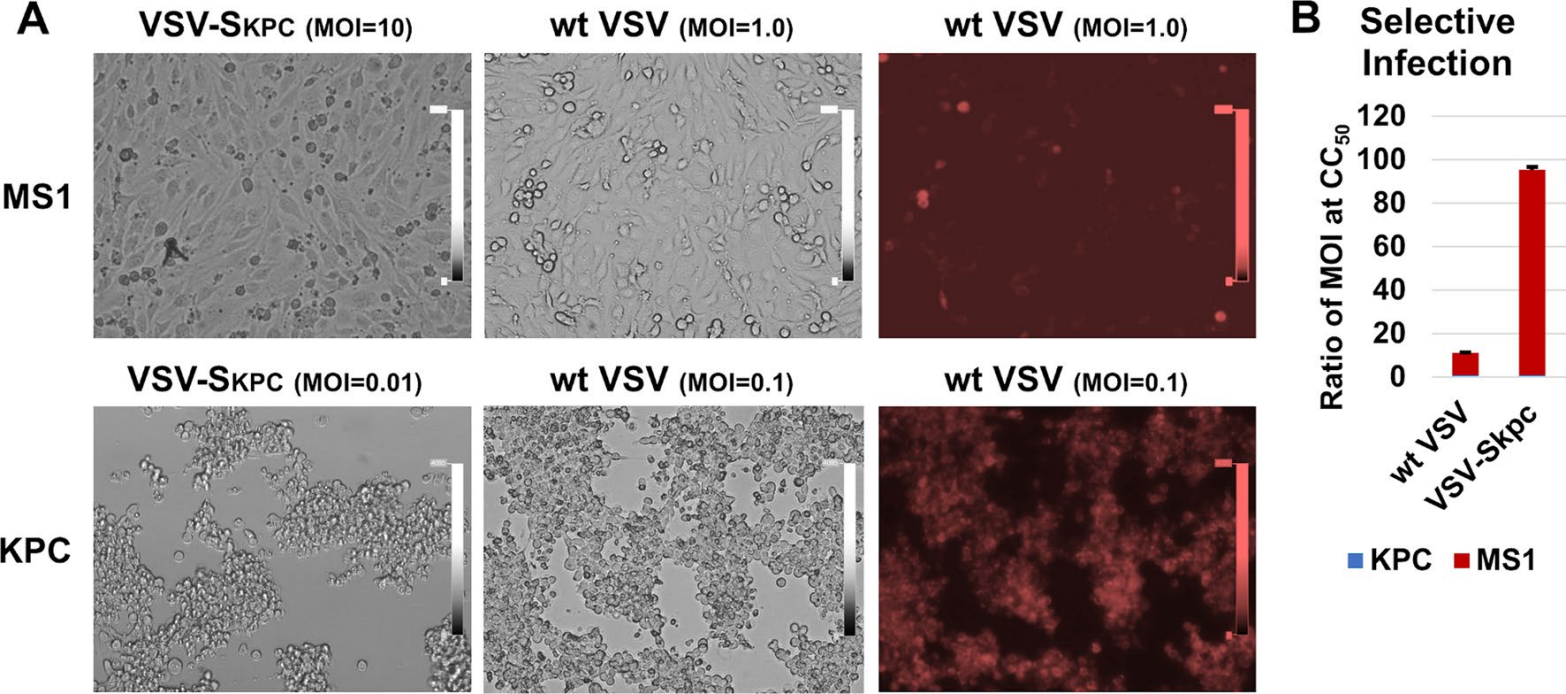
Selective infection. **a** MS1 and KPC cells were infected by VSV-S_KPC_ and wt VSV at MOI from 10 to 0.01. wt VSV expresses a red fluorescent P protein during infection (right panels). **b** Ratios of MOI for CC_50_ in MS1 cells versus KPC cells. CC_50_ values were determined by MTT assays, and error bars from duplicates were included

To confirm that adaptation by limited dilution increases selective infection by VSV-SKPC, mouse KPC and MS1 cells (pancreatic islet endothelial cell line, ATCC) were infected with VSV-SKPC and wt VSV at different MOIs (Fig. 1a). KPC cells could be effectively infected by wt VSV at MOI = 0.1, and MS1 cells, at MOI = 1. On the other hand, KPC cells could be effectively infected by VSV-SKPC at MOI = 0.01, and MS1 cells, at MOI = 10. The selective ratio is 10:1 (KPC:MS1) for wt VSV based on CC_50_ (the MOI value required to kill 50% of cells) measured by MTT assays (Fig. 1b), whereas the selective ratio is 94:1 (KPC:MS1) for VSV-SKPC. This confirmed that selective infection of VSV-S was increased by adaptation by limited infection.

### Preparation of concentrated VSV-S

For tumor treatment, a dose of concentrated VSV-S would be injected intratumorally. Since VSV-SKPC has an increased infectivity of KPC cells, it was propagated in KPC cells. This is the first time that large quantities of VSV were grown in KPC cells. Large plates (150 mm diameter) of KPC cells were cultured with DMEM supplemented with 10% fetal bovine serum (FBS). When cells were grown to confluence, the culture media were removed and washed once with warm Dulbecco's phosphate-buffered saline. Cells were infected at MOI = 0.1 and the culture media of the infected cells were harvested in 48 h. The virus was concentrated by ultracentrifugation at 23,400 g for 2 h. The virus pellet was resuspended in PBS containing 5% sucrose and stored in liquid nitrogen for later animal studies. The titer of the concentrated virus sample was 1.0 ± 0.1 × 10^9^ PFU/mL determined in HeLa cells, which is consistent with the fold of sample volume reduction. The concentrated VSV-SKPC was used in all the animal studies.

### Changes of the TME induced by VSV-S

**Fig. 2:**
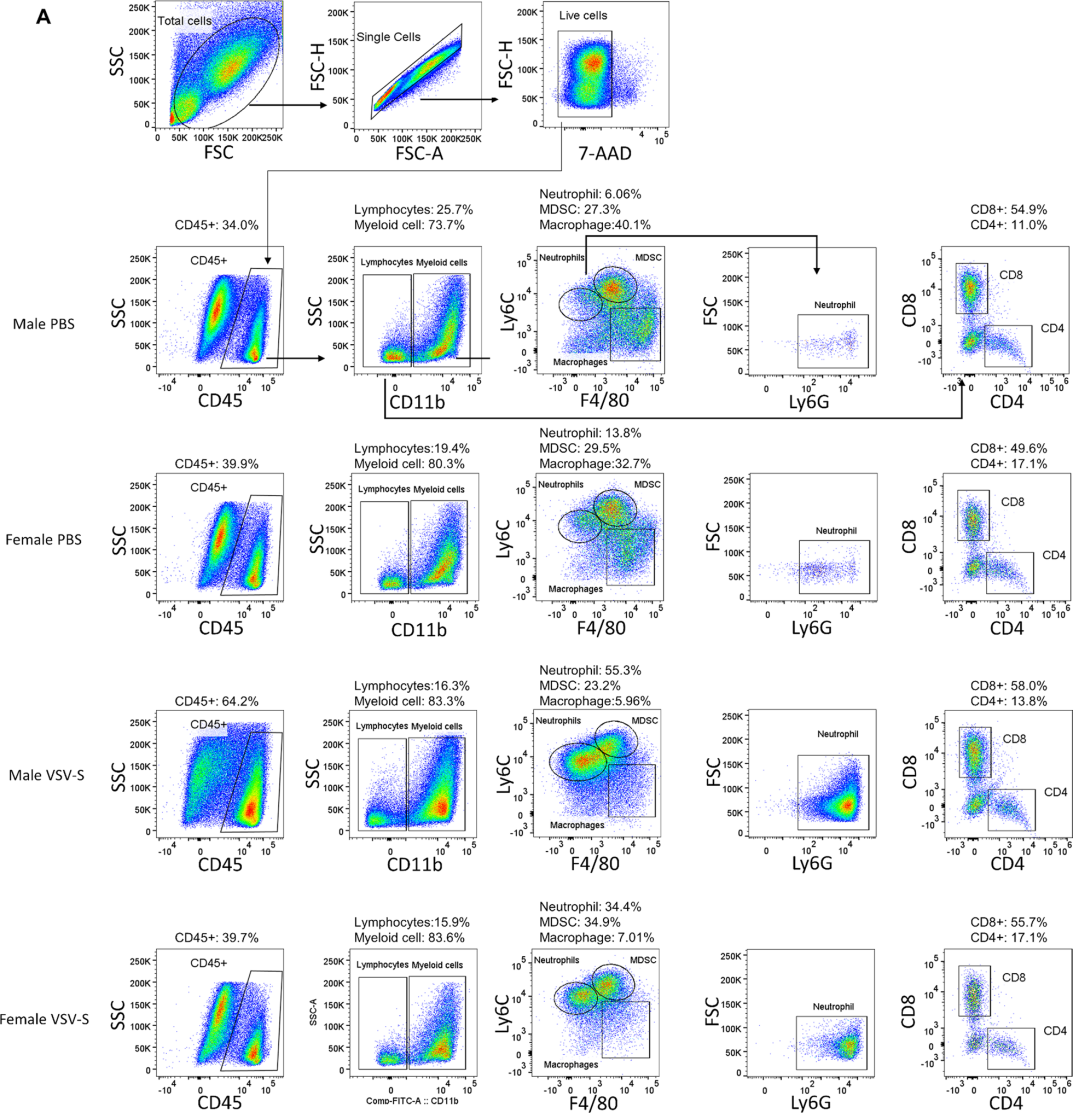

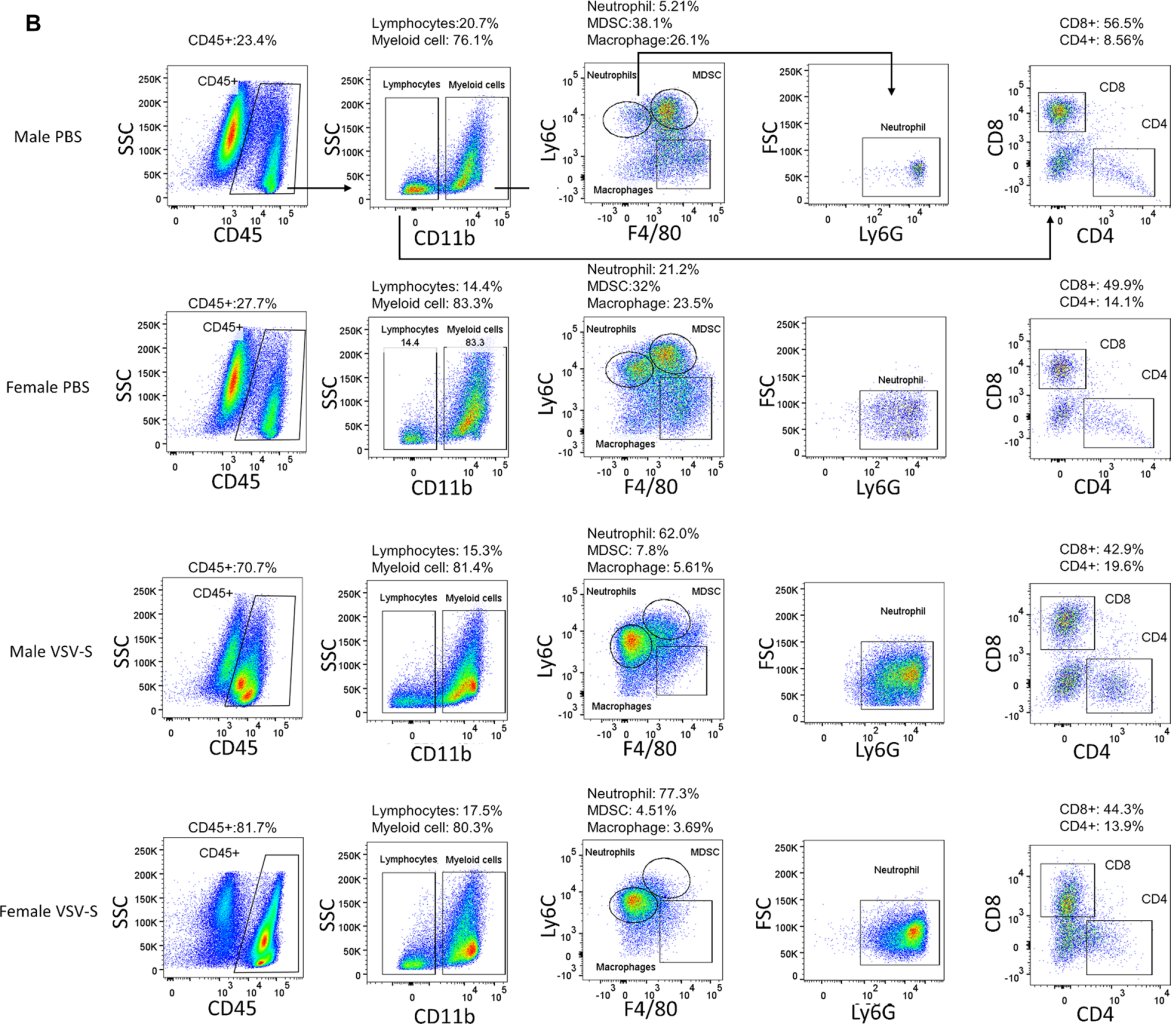

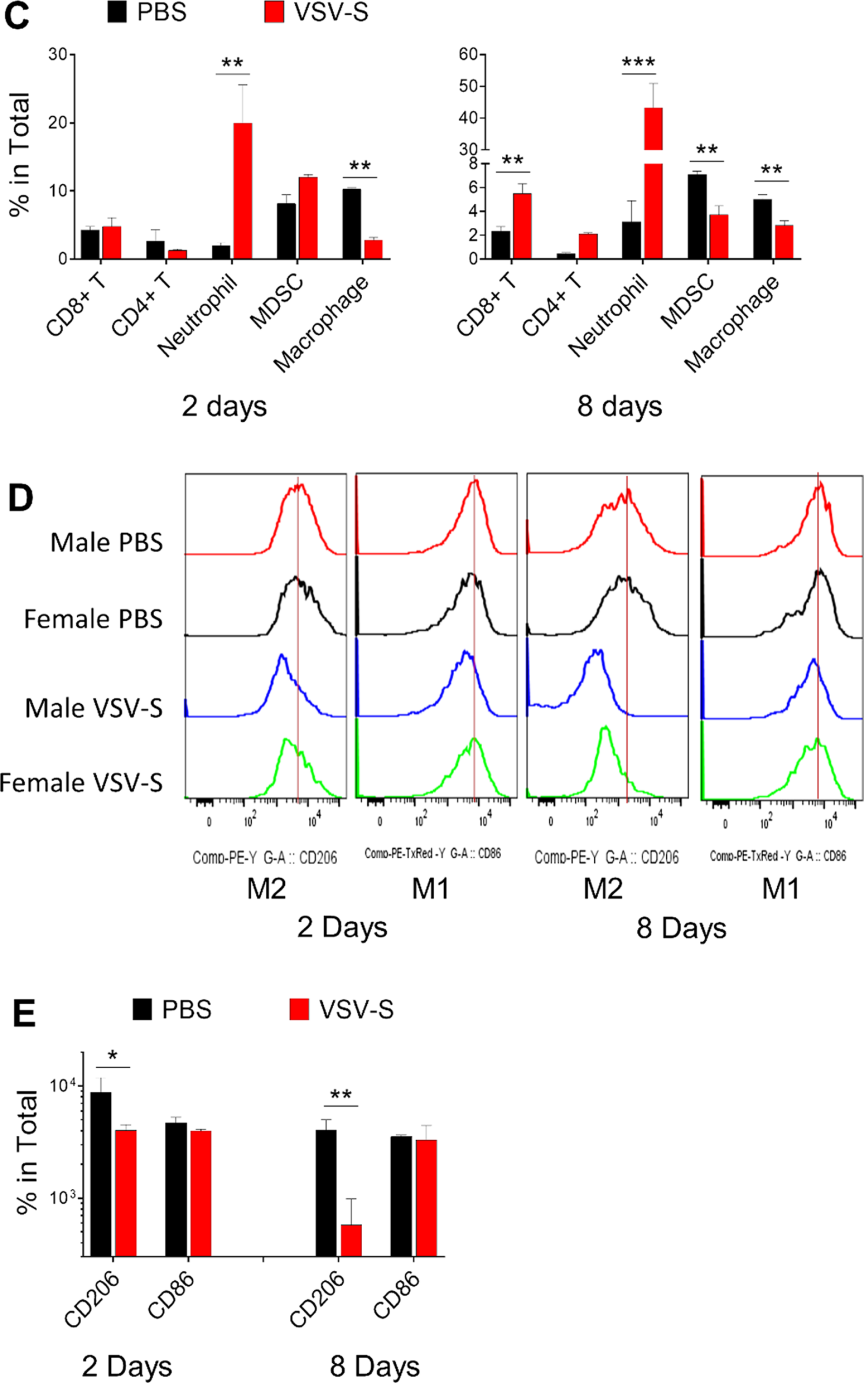

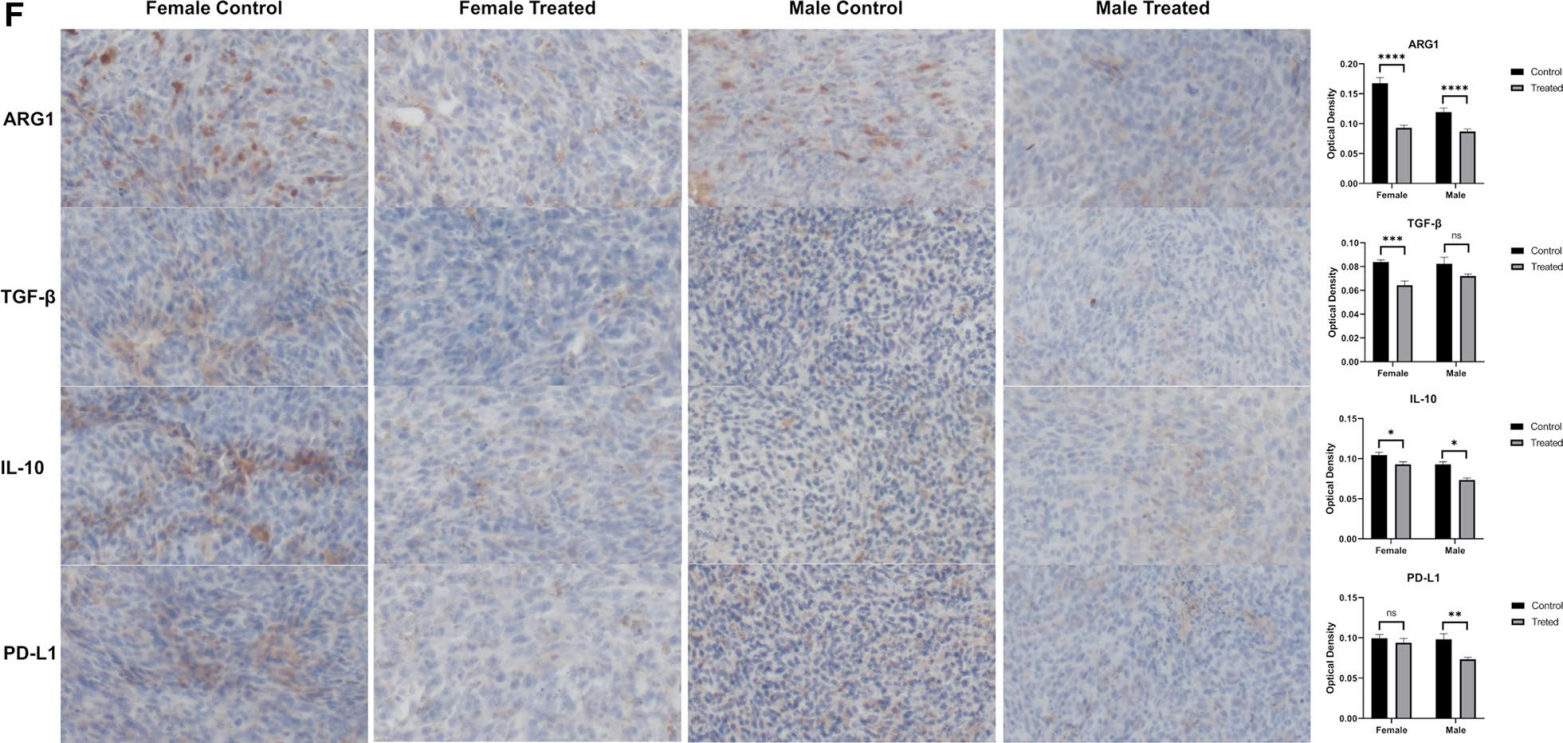
Analyses of the TME. **a** Gating strategy for flow cytometry and analyses of cells isolated from tumors in representative male and female mice 2 days (Day 6) after treatment with intratumoral injection of VSV-S or PBS control. Gates and biomarkers are labelled. Arrows point out the relationship between the panels. **b** Same as in (**a**) from mice 8 days (Day 12) after treatment. **c** Quantitative comparisons of leukocytes on Days 6 and 12 after VSV-S treatment. Statistical methods are discussed in Materials and Methods. **d** Sorting of macrophages by CD206 + (M2) or CD86 + (M1) markers in tumors in male and female mice 2 (Day 6) or 8 (Day 12) days after treatment. Color codes are: red, male PBS; black, female PBS; blue, male VSV; and green, female VSV. Quantitative comparisons of data from each mouse are presented in the bar charts. **e** Quantitative comparisons of macrophages on Days 6 and 12 after VSV-S treatment. Statistical methods are discussed in Materials and Methods. (F) Comparison of the tumor IHC staining between VSV-S treated and PBS control (Day 12) mice. Comparisons of average values from 10 sections from each mouse are presented in the bar charts

To characterize the TME after treatment with VSV-S, syngeneic tumors were established by subcutaneous injection of 0.5 × 10^6^ KPC_Luc cells in the flank of both male and female C57BL/6 mice. Tumors grew to similar visible sizes between 6 and 8 days, and treatment initiation of the grown tumors was set as Day 0. On Days 0, 2, and 4, 3.0X10^7^ PFU of VSV-S_KPC_ in 30 µL or 30 µL of PBS control was intratumorally injected in each mouse. Tumor samples were collected on Days 6 and 12, respectively, after the second injection of VSV-S or PBS control. After dissociating tumor tissues, the immune components in the TME were analyzed. As shown in Fig. 2, the total amounts of tumor-infiltrating leukocytes (CD45 +) appeared to be not significantly changed on Day 6 after VSV-S treatment, but significantly increased on Day 12. This latter change was associated with reduction of the KPC tumor burden. However, the ratio between lymphocytes (CD11b-CD3 +) and myeloid cells (CD11b +), or CD8 + and CD4 + T cells, was not changed significantly. Between lymphoid and myeloid compartments, we found major changes within the latter. Particularly, VSV-S treatment induced large infiltration of neutrophils into tumors, a condition indicative of a strong inflammatory response in the TME. On Day 6, neutrophils in VSV-S-treated tumors were increased by 7–10 folds, occupying over 50% in the total tumor-infiltrating leukocytes (CD45 +), and this phenomenon of neutrophil infiltration continued to Day 12 when the tumor burden was further reduced. Meanwhile, tumor-associated MDSCs and macrophages were largely reduced. These results together suggest that the acute inflammatory response and neutrophil-mediated killing played the primary role in the rapid elimination of KPC cancer resulted from VSV-S infection. In comparison, the control treatment with PBS did not induce neutrophil infiltration but maintained MDSC- and macrophage- centered tumor immunosuppression.

To assess the degree of reverting the immunosuppressive TME, we assessed tumor-associated macrophages for pro- (M1) and anti-inflammatory (M2) phenotypes in the midst of large reduction in the macrophage population. As shown in Fig. 2d and e, macrophages within the VSV-S treated tumors exhibited significantly reduced expression of M2 phenotype marker (CD206 +) on both Day 6 and Day 12, whereas macrophages in control tumors maintain high expression of the M2 phenotype. These observations suggest the treatment of VSV-S induced macrophage phenotypic changes to reshape the TME towards proinflammatory direction that enhances the antitumor immune response.

To further validate the changes of the TME caused by the VSV-S treatment, immunohistochemistry staining of the paraffin-embedded tumor tissues was also performed. Immunosuppressive factors arginase1 (ARG1), transforming growth factor beta (TGF-β) and interleukin 10 (IL-10) were significantly decreased in tumors from both male and female mice treated with VSV-S compared to those in tumors of the mice treated with vehicle. These data were consistent with the notion that VSV-S treatment has favorable immune modulatory effects in the TME (Fig. 2f). The PD-L1 expression level was also decreased in tumors 8 days after treatment with VSV-S (Fig. 2f). Previously published data showed that the increased expression of tumor PD-L1 was localized primarily to myeloid cells [27]. The observed decrease of PD-L1 expression after VSV-S treatment may be associated with the decrease of myeloid cells shown in Fig. 2b.

### Inhibition of KPC tumor growth

**Fig. 3:**
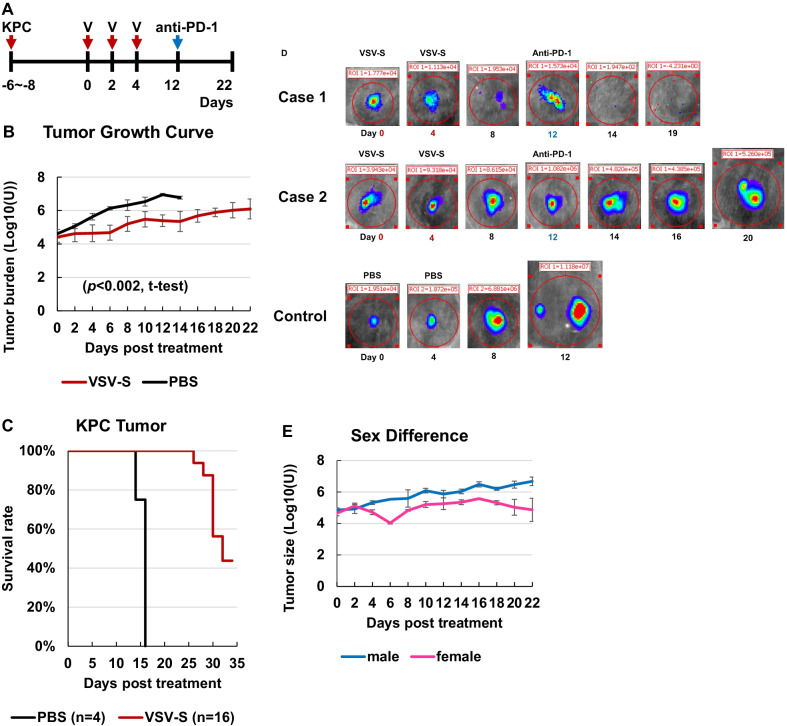
**a** Scheme of tumor implant (KPC), and treatment with VSV-S (V) and anti-PD-1 antibody. **b** Tumor growth curves. The tumor growth curves were plotted for VSV-S treated (n = 16) (red) and PBS control (n = 4) (black) groups. Tumor burden was presented as logarithm values of the luciferase activity measured on an IVIS imager. Error bars represent the standard deviations. Treatment regimen was intratumoral injection of 3.0 × 10^7^ PFU of VSV-S_KPC_ on Days 0, 2 and 4, and 40 µg of anti-PD-1 antibody on Day 12. The control was intratumoral injection of 30 µL of PBS on Days 0, 2 and 4. (*p* < 0.002, t-test) **c** Survival of treated and control mice. The survival rate of mice in the treated group (red) and the control group (black) was plotted versus days post treatment. The treatment regimen and control are the same as in (ii). **d** Tumor growth patterns. IVIS images of luciferase activities from three examples are presented. Days of virus, PBS and anti-PD-1 antibody injections are marked. Case 1 is for a female mouse that resulted in complete regression of the tumor after treatment, whereas Case 2 is for a female mouse that resulted in extended inhibition of tumor growth after treatment. A case for PBS control is also included. **e** Sex difference. The tumor growth curves of treated female mice (n = 3) (pink) and male mice (n = 3) (blue) groups. Tumor burden was presented as logarithm values of the luciferase activity measured on an IVIS imager. Error bars represent the standard deviations. Treatment regimen was intratumoral injection of 3.0 × 10^7^ PFU of VSV-S_KPC_ on Days 0, 2 and 4, and 40 µg of anti-PD-1 antibody on Day 12. (*p* < 0.0008, t-test)

Previously, our study showed that VSV-S has an enhanced antitumor activity in comparison with wt VSV [23]. The experiment here was to examine the antitumor activity of adapted VSV-S and the consequence of TME changes on the effect of anti-PD-1 therapy. Syngeneic tumors were established as in 1.3. When tumors became visible, the tumor burden was measured as the integrated luciferase activity via IVIS imaging. The treatment regimen is outlined in Fig. 3a. On Days 0, 2, and 4 following confirmation of established tumors, 3.0X10^7^ PFU of VSV-S_KPC_ in 30 µL was intratumorally injected in each mouse, and tumor growth was recorded every 2 days until Day 22 (Fig. 3b). For controls, 30 µL of PBS was intratumorally injected according to the same schedule. On Day 12, 40 µg of anti-PD-1 antibody was intratumorally injected in each of the VSV-S treated mice. The tumor growth curves and the rate of survival are presented in Fig. 3b and c. Tumor burdens were monitored by measuring the luciferase activities using an IVIS imager. The quantitation is 4.5 × 10^6^ U/g of tumor mass.

Tumor growth was inhibited statistically more substantial by treatment with VSV-S in comparison with the control (*p* < 0.002, t-test) (Fig. 3b). In some cases, VSV-S treatment effectively arrested tumor growth (e.g. Case 1 in Fig. 3d). The followed-up treatment with anti-PD-1 antibody led to complete regression of the tumor. However, complete regression of the tumor was not achieved in cases exemplified by Case 2 in Fig. 3d, despite obvious inhibition of tumor growth by VSV-S treatment as well as the follow-up treatment with anti-PD-1 antibody. The most definitive beneficial outcome of VSV-S treatment, followed by treatment with anti-PD-1 antibody, is that the survival of the tumor bearing mice was significantly increased (Fig. 3c). 7 mice survived beyond 35 days and were tumor-free after the treatment. The average size of the tumors at the initiation of VSV-S injection was 4.01 ± 0.26 log10 (U) of the luciferase activity for the 7 survived tumor-free mice, compared to 4.72 ± 0.25 log10 (U) for the other 9 mice. The mean-times to death (MTD) is doubled to > 27.4 days compared to MTD of 14.9 days for the control group. Interesting differences in tumor growth were found between female and male mice (*p* < 0.0008, t-test) (Fig. 3e). The tumor growth rate is faster in male than in female mice, with or without treatment with VSV-S.

### Safety data of VSV-S in mice

Blood biomarkers were measured as a surrogate for safety in mice with and without subcutaneous tumors. 1.0 × 10^8^ PFU VSV-S in 100 µL was administered in C57BL/6 mice via tail vein injection. After 24 h, blood samples were collected from the mice and were subjected to clinical chemistry, hematology and coagulation analyses. The data are summarized in Table 3.

**Table 3:** Safety data from VSV-S treatment

	Tumor bearing mice (n = 2)	Mice w/o tumor (n = 2)
*Hematology*		
WBC (10^3^/µL)	–^a^	22.6
RBC (10^3^/µL)	3.2	9.8
HGB (g/dL)	4.8	14.7
HCT (%)	16.7	48.4
MCV (fL)	52.0	50.5
MCH (pg)	14.9	15.4
MCHC (g/dL)	28.7	30.5
Platelet (10^3^/µL)	74	282
Lymphocytes (%)	34.0	87.5
Neutrophil (%)	52.0	8.0
Band (%)	0	0
Monocytes (%)	13.0	4.5
Eosinophils (%)	1.0	0
Basophils (%)	0	0
*Clinical chemistry*		
Hemolysis	+++	++++
Lipemia	Normal	Normal
ALT (U/L)	277.0	247.5
ALP (U/L)	6.0	32.0
Albumin (g/dL)	2.3	3.5
Total protein (g/dL)	4.6	6.3
Glucose (mg/dL)	92.0	78.0
Calcium (mg/dL)	8.4	8.4
Phosphorus (mg/dL)	8.7	15.1
BUN (mg/dL)	28.0	26.5
PTT (s)	> 70	> 70

^a^No countable data obtained

## Discussion

Methods that turn PDAC from immunologically “cold” to immunologically “hot” are likely to improve the outcome of immunotherapy such as immune checkpoint blockade. Among numerous approaches, treatment with OV may have some unique advantages. Infection of tumor cells by OV can stimulate inflammatory responses in the TME and release quantities of tumor antigens to elicit anti-tumor immunity. A number of OVs have been evaluated in Phase II trials, including adenovirus (ONYX-015) [28], reovirus (Reolysin) [29] and parvovirus (ParvOryx) [30]. While treatment with OVs, or in combination with gemcitabine, was well tolerated, the positive outcomes are encouraging in some cases, but not clearly conclusive in the overall study [31].

To design a robust OV that potently targets cancer cells, we constructed VSV-S derived from a rapidly replicating virus, vesicular stomatitis virus. VSV expressing INFβ (VSV-IFNβ-NIS) has been investigated in a phase I-II study in patients with refractory solid tumors, showing a good safety profile, and is currently evaluated in combination with cemiplimab [32]. In our study, VSV-S expresses the Smac protein, a pro-apoptotic protein, during its infection. Our previously published data have shown that VSV-S maintains a high level of intracellular Smac and induced a high degree of apoptosis in VSV-S infected cells, whereas infection by the wt VSV diminished endogenous Smac and has a more prominent resistance by several cancer cell lines [23]. In addition to the robustness of VSV-S in killing cancer cells, we developed here a strategy to enhance cancer targeting of VSV-S through adaptation by limited dilution. VSV has a broad cell tropism [25], which permits sufficient initial infectivity in the selected target cell. Since VSV replicates rapidly in most cells, targeting the selected cell can be achieved through adaptation, an efficient approach commonly used for increasing virus selectivity [33]. Other groups have adapted oncolytic VSV to improve replication efficiency (i.e. virus yield) [34]. Our adaptation strategy is using limited dilution to increase selective infection, rather than a high virus yield. In each round of infection, the inoculum virus was selected from the supernatant of cells infected with the highest dilution in the previous round. By a few rounds of adaptation under such a strategy, the selective infectivity of VSV-S was increased by 100-fold in MIA PaCa-2 cells (Table 1), a human PC cell line, and 20-fold in mouse KPC cells (Table 2). To directly confirm the selective infectivity, infection of KPC and MS1 cells by adapted VSV-S was compared by their CC_50_ values measured with MTT assays (Fig. 1). The selectivity is 94 fold for KPC cells over MS1 cells. Our results demonstrated that VSV-S adaptation by limited dilution is readily applicable to target other cancer cells in a very short period of time (a few days).

The ultimate application of VSV-S or its derivatives is to enhance the efficacy of treatment for PDAC and other cancers. Previously, we have shown that VSV-S has a superior antitumor activity in animal models in comparison with wt VSV [23]. Published animal studies also confirmed that the efficacy of anti-PD-1 therapy alone is very limited [35–38]. Our primary interest is to optimize the combination therapy of OV and checkpoint inhibitors, especially when adapted VSV-S changed the TME significantly. The study using the syngeneic mouse model for PDAC was carried out to investigate adapted VSV-S. Overall, intratumoral injection of VSV-S_PKC_ steadily inhibited tumor growth, occasionally tumors were eliminated (2 out of 8 female mice). Treatment with anti-PD-1 antibody, 8 days after virus injection, prolonged inhibition of tumor growth or even helped to eliminate the tumor (3 out of 8 female mice, and 2 out of 8 male mice). Although we did not include an anti-PD-1 antibody-only group as in previously published reports, treatment by anti-PD-1 antibody alone did not change tumor growth or survival in KPC mouse model as reported in [39] and survival was increased by combination therapies [36]. The delayed administration of the immune checkpoint inhibitor in combination with OV is consistent with the temporal change of the TME, and is cooperated with results from similar studies [40]. The combination therapy greatly extended the survival of tumor bearing mice, achieving a long-term survival of 44% (7 out of 16 subjects).

The efficacy of the treatment with OV appeared to be dependent on the initial tumor size when OV treatment began. This could be due to the degree of virus spread within the tumor mass. When the tumor size is smaller, it is expected that a higher portion of the tumor would be directly exposed to OV infection upon intratumoral injection. It is also observed that the growth rate of tumors in female mice after treatment was significantly slower than that in male mice. The responses to VSV-S/anti-PD-1 treatment are also more favorable in female mice versus male mice. It is not clear what the observation really means in this limited study. The patient numbers of incident cases and deaths peaked at the ages of 65–69 years for males and at 75–79 years for females [41]. PDAC appears to be more devastating to males than females. It might be possible that our preliminary data could suggest that female patients may respond better to virotherapy.

To achieve tumor regression, the rate of cancer killing by OV and TIL must overtake that of tumor growth. In this subcutaneous model, treatment with OV modestly reduced the rate of tumor growth. More importantly, however, treatment with OV altered the TME that could enhance the efficacy of immunotherapy. This advantage is illustrated by large changes of leucocytes in the tumors infected by VSV-S (Fig. 2). The change was noticeable after 2 days, and more pronounced after 8 days, following the final VSV-S infection. Despite that the total number of lymphocytes was greatly increased, the ratio between lymphocytes and myeloid cells, or between CD8 + and CD4 + T cells, however, was not changed significantly. The most prominent changes of myeloid cell compartments were the dramatic increase of neutrophils, and the decrease of MDSCs and macrophages. These changes are consistent with strong inflammation in the tumor caused by VSV-S infection. Neutrophils exhibit tumor suppressive activities by generating reactive oxygen species (ROS), activation of the IFN-γ pathway, and up-regulation of antigen presentation [42]. The large increase of neutrophils in tumors could cause more death of cancer cells due to innate immunity [43]. Several studies confirmed that neutrophils participated in tumor cell clearance upon OV infection of tumors [44–47], which is also consistent with our previous results of efficacious tumor regression by VSV-S treatment of breast cancer xenografts [23].

The large reduction of macrophages, especially M2-like macrophages (Fig. 2e, f), most likely were related to reverting the immunosuppressive TME. To confirm this notion, levels of ARG1, TGF-β, and IL-10 in tumors were analyzed by immunohistochemistry (Fig. 2g). The association of their expression levels with the immune conditions in the TME is not always clear cut. For instance, overexpression of arginase is perceived as a poor prognostic factor in a wide variety of cancer types [48]. Myeloid cells are major contributors to immune defense against pathogens and play an important role in tissue remodeling. In the tumor, myeloid cells are highly heterogeneous. Cells associated with strong immunosuppressive functions are mainly MDSCs that express arginase. It has been shown that arginase plays an opposite role in the immune response and is one of the main mechanisms of immunosuppression [49]. MDSCs also express suppressive cytokines like TGF-β and IL-10 in the tumor [49]. Our observation is consistent with changing the immunosuppressive TME by VSV-S infection of the tumor. The observed reduction in PD-L1 may be associated with its reduction in myeloid cells, not necessarily PD-L1 expressed in tumor cells. More extended regression of tumors should be achievable by optimal combination regimens of OV treatment and immunotherapy.

Preliminary data of clinical chemistry, hematology and coagulation were collected in tumor bearing and control mice after 1.0 × 10^8^ PFU of VSV-S_KPC_ was intravenously injected (Table 3). There is no indication of safety concerns. Our data are similar as those obtained for another modified VSV [50]. Treatment with VSV-S increased white blood cells (WBC) in mice without tumors. Lymphocyte counts in tumor bearing mice were reduced, whereas they are normal in control mice. Neutrophils and monocytes were largely increased in tumor bearing mice than mice with tumors. The changes in these cells are due to tumors in mice, not treatment of VSV-S. No other significant side effects were observed.

## Conclusion

Our results demonstrated that infection by VSV-S dramatically changed the immune cell distribution in the TME. The TME changes are consistent with the beneficial outcome of combination therapy with anti-PD-1 antibody. More detailed immunological studies may be extended from the current data to further optimize the combination therapy in orthotopic PDAC models.

## Data Availability

All data are available upon request.

## Abbreviations

PDAC: Pancreatic ductal adenocarcinoma
KRAS: Kirsten-ras protein
CDKN2A: Cyclin-dependent kinase inhibitor 2A
TP53: Tumor protein p53
SMAD4: Mothers against decapentaplegic homolog 4
5-FU: 5-Fluoruracil
LV: Leucovorin
TME: Tumor microenvironment
IL-6: Interleukin-6
TGF-β: Transforming growth factor-β
Treg: T regulatory cells
MDSC: Myeloid derived suppressor cells
OV: Oncolytic virus
TAM: Tumor-associated macrophages
scRNA-seq: Single cell RNA sequencing
FACS: Fluorescence-activated cell sorting
VSV: Vesicular stomatitis virus
IAP: Inhibitor of apoptosis proteins
CPE: Cytopathogenic effect
FBS: Fetal bovine serum
MTD: Mean-times to death
WBC: White blood cells
